# Mechanisms and therapeutic strategies of macrophages and neutrophils inducing ulcerative colitis progression

**DOI:** 10.3389/fimmu.2025.1615340

**Published:** 2025-08-29

**Authors:** Haogeng Wang, Taixi Huang, Yuxia Ma

**Affiliations:** ^1^ School of Acupuncture and Tuina, Shandong University of Traditional Chinese Medicine, Jinan, China; ^2^ Key Laboratory of Traditional Chinese Medicine Classical Theory, Ministry of Education, Shandong University of Traditional Chinese Medicine, Jinan, China

**Keywords:** ulcerative colitis, neutrophil extracellular traps (NETs), macrophage polarization, mucosalimmunity, biomarkers, targeted therapy

## Abstract

Ulcerative colitis (UC) is a kind of chronic inflammatory bowel disease, is driven by dysregulated immune responses involving neutrophils (NEUs) and macrophages. NEUs exacerbate mucosal injury through reactive oxygen species (ROS), neutrophil extracellular traps (NETs), proteases, and cytokine interactions, while also exhibiting dual roles in tissue repair. Macrophages contribute to UC progression via M1-mediated pro-inflammatory cytokine release and epithelial barrier disruption, whereas M2 macrophages promote resolution through anti-inflammatory signals (IL-10, TGF-β) and epithelial regeneration. Clinically, NEU-derived biomarkers predict disease activity and therapeutic response, while macrophage-targeted therapies modulate inflammation. This review summairzes current knowledge on the mechanistic roles of these immune cells in UC pathogenesis and their clinical implications, such as NET inhibition, MMP-9 blockade, and M2 polarization, which hold promise for precision medicine in UC.

## 1 Introduction

Ulcerative colitis (UC), a major subtype of inflammatory bowel disease (IBD), is characterized by chronic, relapsing inflammation of the colorectal mucosa, leading to bloody diarrhea and abdominal pain, which may be life-threatening in severe cases (1). Since the early 21st century, UC has emerged as a global health concern, with rising prevalence imposing a significant socioeconomic burden (2). Therapeutic goals have evolved from clinical to endoscopic and now histological remission, as persistent histologic inflammation despite endoscopic healing is linked to poorer prognosis (3).

The pathogenesis of UC is multifactorial, involving gut microbiota dysbiosis, disruption of the intestinal mucosal barrier, and aberrant immune cell function (4). Among these immune elements, neutrophil (NEU) infiltration is a defining histological feature of UC, with NEU depletion associated with lower relapse risk, and NEU-related biomarkers offering prognostic value (5). Macrophages are essential for phagocytosis and immune modulation. Studies have demonstrated that the number of macrophages in the lamina propria of the colon in patients with active UC is approximately tenfold higher than that in healthy individuals (6), and skew toward a more activated state (7, 8), suggesting their pivotal involvement in UC pathogenesis. This review aims to provide a comprehensive overview of the mechanistic roles of neutrophils and macrophages in the development and progression of UC, as well as their potential clinical applications.

## 2 NEUs regulate the intestinal inflammation of UC

### 2.1 NEU-derived ROS and NETs exacerbate intestinal inflammation

**Figure 1:**
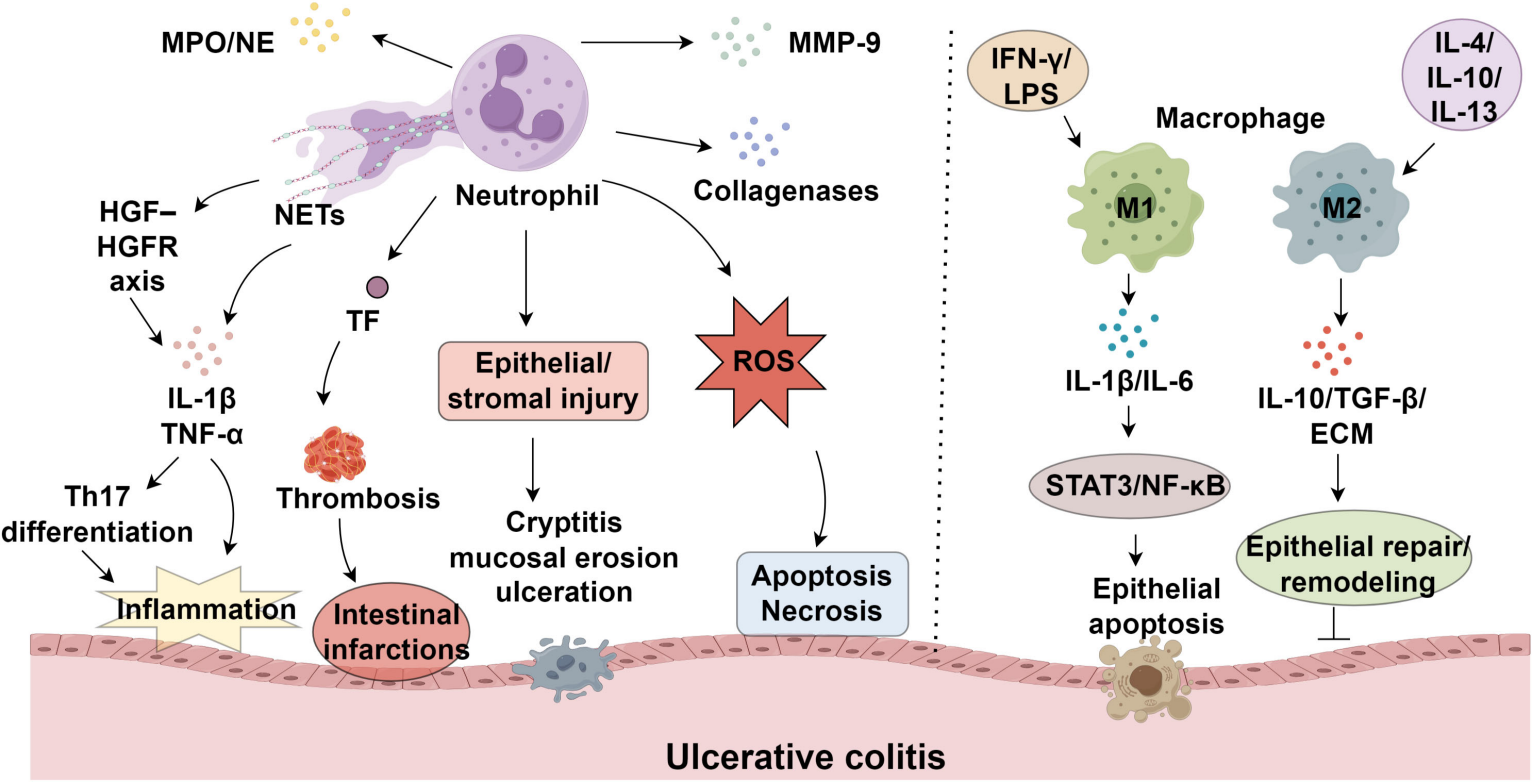
Neutrophils and macrophage polarization in ulcerative colitis progression.

NEUs are essential effectors of innate immunity, yet their excessive activation has been implicated in the onset and progression of various autoimmune diseases (9, 10). During maturation, NEUs generate three distinct types of granules: primary granules, which contain enzymes such as myeloperoxidase (MPO) and neutrophil elastase (NE); secondary granules, including collagenases; and tertiary granules, which carry MMP-9 (11). In UC, massive infiltration of NEUs into the intestinal mucosa leads to the release of granule contents and ROS, resulting in epithelial and stromal injury and manifesting as cryptitis, mucosal erosion, and ulceration (12). ROS induces cellular apoptosis and necrosis by oxidatively damaging nucleic acids, proteins, and lipids (13). In UC, excessive ROS production by infiltrating NEUs, coupled with insufficient ROS clearance, leads to ROS accumulation in the mucosa (14, 15). NETs are extracellular mesh-like structures composed of decondensed chromatin, DNA, and antimicrobial peptides, extruded from activated NEUs as part of their antimicrobial defense (16, 17). NETs amplify inflammatory cascades through the release of IL-1β and TNF-α, representing a key trigger of immune dysregulation in UC (18). Angelidou et al. demonstrated that activation of the REDD1/autophagy/NETs/IL-1β axis mediates UC-related inflammation and mucosal injury (19). Moreover, UC is a recognized risk factor for venous thromboembolism, including deep vein thrombosis and pulmonary embolism (20). NEUs also secrete proteinase-3 and cathepsin G, while NE specifically degrades extracellular matrix components such as elastin (21, 22). Serine protease inhibitor B1, an endogenous NE suppressor, inhibits H_2_O_2_-induced NE activity and may help preserve epithelial integrity (23). Infliximab, a TNF-α-targeting monoclonal antibody and the first biologic approved for moderate-to-severe UC, effectively induces mucosal healing (24) (**Figure 1**).

### 2.2 Cytokine–NEU interactions drive inflammatory activity

Matrix metalloproteinases (MMPs), a family of zinc-requiring endopeptidases, play critical roles in extracellular matrix degradation and tissue remodeling, with their overexpression implicated in immune-mediated tissue damage (25, 26). Within ulcerative colitis, these enzymes drive disease progression through multiple mechanisms, including basement membrane breakdown, enhanced barrier permeability, regulation of epithelial repair, leukocyte migration, and angiogenic modulation (27, 28). Among MMPs, MMP-9 is predominantly secreted by NEUs upon degranulation and serves as a key contributor to UC pathogenesis (29, 30). By compromising epithelial tight junction integrity, MMP-9 exacerbates mucosal permeability and impairs barrier function (31). During active UC, NEUs constitute the predominant immune cell population in the lamina propria, acting as major effectors of mucosal injury (32). The dynamic interaction between NEUs and inflammatory cytokines is fundamental to UC development. Circulating and tissue-infiltrating NEUs produce IL-1β, which amplifies inflammatory responses and tissue destruction via dual mechanisms: NEU-derived serine proteases and inflammasome/caspase-1 activation (33). Hence, targeting NEU serine proteases or caspase-1 may offer novel therapeutic strategies. Stakenborg et al. reported that NEUs promote IL-1β and TNF-α production via the HGF–HGFR tyrosine kinase signaling axis, promoting Th17 differentiation and mucosal inflammation (34). Additionally, antigen-primed NEUs contribute significantly to UC exacerbations; upon re-exposure to antigens, IgG-bound Fcγ receptor I engagement on sensitized NEUs induces TNF-α release, further aggravating inflammation and precipitating disease recurrence (35).

### 2.3 Microbiome-NEUs crosstalk

NEUs play a paradoxical role in intestinal pathology, contributing to both inflammatory responses and tissue protection (36) While defending against microbial invasion through phagocytosis, NETs, antimicrobial peptides, and ROS. Simultaneously, NEUs also secrete cytokines, chemokines, and growth factors that facilitate mucosal repair and barrier regeneration (37). Notably, specific NEU subpopulations demonstrate enhanced protective functions. For instance, CD177^+^ neutrophils generate elevated ROS and antimicrobial peptides, strengthening mucosal defense while suppressing pro-inflammatory cytokine expression (38). Furthermore, CD177^+^ neutrophils produce IL-22, a key mediator in maintaining epithelial homeostasis (36). Research by Leppkes et al. revealed that NEUs accumulating in UC lesions form NETs in a PAD4-dependent manner, transforming blood clots into immune thrombi to reduce hemorrhage and accelerate tissue repair (39). The gut microbiota profoundly regulates NEU behavior in UC through multiple molecular mechanisms (40). Bacterial fermentation of dietary fibers yields short-chain fatty acids (SCFAs), including butyrate, propionate, and acetate, which are crucial in controlling neutrophil function (41). By activating GPR41 and GPR43 receptors on NEUs, SCFAs fine-tune ROS generation and facilitate inflammatory resolution (42). However, microbial dysbiosis in UC diminishes SCFA levels, compromising neutrophil regulation and perpetuating chronic inflammation (43). Additionally, microbial components directly influence NET formation. Pathogens such as Escherichia coli and Clostridium difficile induce NET release by engaging pattern recognition receptors (PRRs), particularly Toll-like receptors (TLRs), which initiate downstream signaling cascades (44, 45). Bacterial products like lipopolysaccharides (LPS) intensify this response by potentiating PRR activation, thereby aggravating UC-associated inflammation (46, 47). Although NETs, comprising DNA, histones, and antimicrobial proteins, worsen tissue injury, they also confine pathogens and restrict dissemination (48). Under homeostatic conditions, NETs aid in infection control without inciting persistent inflammation, whereas dysbiosis disrupts this equilibrium, exacerbating mucosal damage and disease severity (49).

### 2.4 Dynamic behavior of NEUs

NEUs occupy diverse functional states along a continuum from immune-enhancing/pro-resolving phenotypes to dysfunctional, hyperinflammatory programs often described as “exhausted” (50). Pro-resolving or immune-enhancing states can be experimentally induced—for example, “resolving memory neutrophils” trained with 4-phenylbutyrate show enhanced antimicrobial functions and distinct transcriptional features, while low-dose endotoxin can reprogram neutrophils toward immune-enhancing phenotypes (51). At the opposite end, chronic or excessive stimulation drives neutrophil programs with sustained inflammatory mediator release and impaired resolution capacity, consistent with exhausted-like states noted in single-cell studies and reviews of IBD myeloid heterogeneity (52, 53). These polarized neutrophil states have concrete implications in UC: immune-enhancing/pro-resolving programs may facilitate epithelial repair and hemorrhage control, whereas dysfunctional/exhausted programs amplify tissue injury through persistent NETosis, protease release, and cytokine production (16, 30, 39). Recognizing and therapeutically steering neutrophils toward immune-enhancing trajectories such as pro-resolving training and cautious innate “training” paradigms while restraining exhausted-like, hyperinflammatory activity could help tailor interventions for patients with persistent histologic activity (54–58).

## 3 Inhibition of NEU in UC

### 3.1 Inhibition of NEU-derived ROS and pro-inflammatory cytokines

Hesperidin methyl chalcone (HMC), a citrus flavonoid derivative, exerts antioxidative, anti-inflammatory, and analgesic effects by enhancing colonic glutathione levels and antioxidant capacity, thereby limiting NEU infiltration and mucosal damage in UC (15). The sesquiterpenoid compound nerolidol (NRD) demonstrates similar protective effects by suppressing myeloperoxidase (MPO) activity, a key marker of NEU recruitment, while concurrently reducing proinflammatory cytokine secretion and colonic inflammation (59). NRD further enhances cellular defense mechanisms through upregulation of superoxide dismutase and catalase, coupled with decreased ROS generation and lipid peroxidation (59–61). Cyclosporine A (CSA), a calcineurin inhibitor used in refractory UC cases, modulates NEU activity via SIRT6/HIF-1α-dependent metabolic regulation, inhibiting ROS production, MPO release, and antimicrobial peptide expression to prevent excessive neutrophil migration and apoptosis (62). Ursolic acid (UA), a triterpenoid isolated from medicinal plants and fruits, effectively reduces epithelial NEU migration and downregulates IL-6 expression in both systemic circulation and colonic tissues (63, 64). The artemisinin-derived compound SM934 exhibits potent immunosuppressive activity by significantly decreasing MPO levels and attenuating macrophage/NEU accumulation in inflamed colonic regions, leading to reduced IL-1β, IL-6, and TNF-α production (65). Another critical regulatory mechanism involves peptidoglycan recognition protein 1 (PGLYRP-1), which stimulates proinflammatory mediator release (TNF-α, IL-1β, IL-6, MPO) from neutrophils upon interaction with triggering receptor expressed on myeloid cells 1 (TREM-1). Therapeutic targeting of TREM-1 with neutralizing antibodies effectively disrupts this pathway, particularly in UC patients exhibiting heightened PGLYRP-1 expression and neutrophil infiltration (66).

### 3.2 Inhibition of NET formation and NE activity

Extrachromosomal DNA (ecDNA) is critically involved in the generation of NETs. The enzymatic degradation of ecDNA within the colonic microenvironment by DNases offers a promising therapeutic strategy for UC (67). To achieve site-specific delivery, staphylococcal nuclease (SNase), a highly efficient phosphodiesterase with broad substrate specificity, was formulated into calcium alginate microspheres (ALG-SNase). This targeted intervention facilitated NET disruption, attenuated inflammatory responses in the colon, enhanced epithelial barrier function, and increased expression of key tight junction proteins, including occludin and zonula occludens-1 (68). Peptidylarginine deiminase 4 (PAD4) is essential for histone citrullination during NET formation. Peptidylarginine deiminase 4 (PAD4) plays a crucial role in mediating histone citrullination, a prerequisite for NET formation. Studies demonstrate that NETs activate the cGAS-STING pathway in MC38 cells in a dose- and time-dependent manner, promoting the release of pro-inflammatory cytokines and impairing intestinal barrier integrity. Genetic ablation of STING ameliorates disease severity, as evidenced by improved clinical colitis scores, reduced intestinal inflammation, and restored barrier function. Notably, suppression of NET generation through PAD4 knockout attenuates STING upregulation (69). Pharmacological inhibition of this post-translational modification has shown therapeutic benefits in UC models (70). However, PAD4-deficient UC mice exhibit impaired mucosal healing due to defective remodeling of fibrin clots at wound sites (39).

Furthermore, NE’s proteolytic activity compromises the TNF-neutralizing efficacy of infliximab, lowering clinical response rates. Co-administration of exogenous protease inhibitors may counteract NE-mediated degradation, enhancing the efficacy of biologic therapy (71). Selective blockade of the neonatal Fc receptor (FcRn) alleviates UC pathology by suppressing NET formation in the colon through enhanced clearance of anti-neutrophil cytoplasmic antibodies (ANCAs) (72). Baicalein (BCL) demonstrates efficacy in preventing UC relapse by downregulating FcRn expression via inhibition of NF-κB signaling mediated by the p50/p65 heterodimer. Prolonged BCL treatment in UC mice significantly reduces colonic FcRn levels, serum ANCA titers, neutrophil-activating peptide (NAP) expression, and inflammatory markers (including TNF-α, IL-1β, and CRP), while improving disease activity indices and histological scores, outperforming sulfasalazine (73).

## 4 The role of macrophages in UC

### 4.1 The role of M1 macrophages in UC

Macrophages exhibit phenotypic plasticity in response to microenvironmental cues, polarizing into pro-inflammatory (M1) or anti-inflammatory (M2) subsets (74, 75). Polarization toward the M1 phenotype is predominantly induced by IFN-γ, LPS, and TNF-α (76). In UC, compromised intestinal epithelium permits microbial invasion, which is detected by M1 macrophages. These cells subsequently overproduce inflammatory cytokines and chemokines (77), exacerbating inflammation, tissue damage, and impaired healing (78, 79), and driving disease progression through cytokine-dependent mechanisms. In contrast, M2 macrophages, stimulated by IL-4, IL-10, or IL-13, exhibit diminished reactivity to bacterial antigens while maintaining phagocytic and antimicrobial activity (80). Their impaired regulatory function contributes to epithelial barrier dysfunction, a key feature of UC pathology (81). Notably, M1 macrophages impair mucosal integrity via excessive MMP secretion, especially MMP-9, which disrupts the ECM, elevating gut permeability and permitting additional immune cell migration (82). Pro-inflammatory cytokines such as IL-1β and IL-6 predominantly released by M1 macrophages (83, 84). Elevated IL-1β levels in UC patients weaken the intestinal barrier, permitting immune cell influx into the lamina propria and aggravating epithelial injury, thereby accelerating disease initiation (85). Similarly, IL-6 exacerbates mucosal edema, increases epithelial permeability, and triggers NF-κB signaling through STAT3 activation, fostering cytokine imbalance and amplifying tissue damage in UC (86). Collectively, these mechanisms sustain chronic inflammation and perpetuate UC progression by undermining intestinal barrier function (**Table 1**).

**Table 1:** Mechanisms and therapeutic strategies targeting neutrophils and macrophages in ulcerative colitis.

Immune Crosstalk	Mechanistic Role	Cytokines & Factors	Roles	Potential Implications
Neutrophils (NEUs)	Exacerbate mucosal damage via ROS production and NET formation. Release proteases (e.g., MPO, MMP-9) that degrade ECM.	IL-1β, TNF-α, IL-6, ROS, MMP-9, NETs	Inhibition of NETs (e.g., DNase therapy), MMP-9 inhibition, NE protease inhibition	NEU-derived biomarkers to monitor disease activity, Targeted therapies for ROS and NET inhibition.
Macrophages (M1)	Release pro-inflammatory cytokines, contributing to tissue damage and inflammation. Polarize towards M1 phenotype in response to IFN-γ, TNF-α, LPS.	IL-1β, IL-6, TNF-α, MMP-9, ROS	M1 polarization inhibition, IL-1β/IL-6 blockade, MMP-9 inhibition	Targeting M1 macrophages may alleviate excessive inflammation in UC.
Macrophages (M2)	Facilitate tissue repair via anti-inflammatory cytokines and ECM remodeling. Induced by IL-4 and IL-13.	IL-10, TGF-β, HGF, FPR/annexin A1, NOX1	M2 polarization induction, IL-10/TGF-β modulation	Inducing M2 macrophage polarization could promote mucosal healing and repair.
Neutrophil-Macrophage Crosstalk	NEU-derived cytokines and NETs influence macrophage polarization towards pro-inflammatory M1 phenotype.	IL-1β, TNF-α, IL-6, MMP-9, TGF-β, IL-10	Targeting NEU-Macrophage interactions (e.g., cytokine and NET inhibition)	Combined therapies targeting both NEUs and macrophages can improve UC management.
Microbiome-NEU Crosstalk	Dysbiosis impairs NEU function, leading to sustained inflammation. SCFAs modulate NEU activity and reduce inflammation.	SCFAs, LPS, TLRs, ROS	Microbiome modulation through probiotics, SCFA supplementation	Restoring microbiome balance may improve NEU function and reduce UC inflammation.

### 4.2 Exacerbation of intestinal inflammation

Under normal physiological conditions, macrophages in the colonic lamina propria express high levels of CX3CR1. However, in UC, microbial invasion or epithelial barrier disruption leads to the recruitment of inflammatory macrophages expressing intermediate CX3CR1 levels (CX3CR1^int^), derived from circulating CX3CR1^low^ Ly6C^high^ CCR2^+^ monocytes. These macrophages produce substantial pro-inflammatory mediators, drive local inflammation, and enhance effector T cell functions (87). Compared to their counterparts in healthy colonic tissue, macrophages in UC exhibit both phenotypic and functional alterations. Macrophages infiltrating inflamed colonic tissue in UC patients display an activated phenotype, increased TNF-α secretion, and enhanced stimulation of mucosal T cells, which, in turn, produce elevated IFN-γ levels (88). This cytokine interplay promotes epithelial apoptosis, compromises the mucosal barrier, and initiates pathological immune responses, leading to further infiltration of activated macrophages and T cells into the colonic mucosa. The disruption of Th1/Th2 homeostasis ultimately sustains and intensifies mucosal inflammation (89).

### 4.3 M2 macrophages in UC

Polarization of M2 macrophages is induced by the cytokines IL-4 and IL-13. In patients with IBD, M1-associated markers and pro-inflammatory cytokines are typically elevated, whereas M2-associated markers and IL-1 (90). In a dextran sulfate sodium (DSS)-induced murine model of UC, upregulation of Yes-associated protein (YAP) in macrophages was shown to drive M2 polarization and increase the production of anti-inflammatory cytokines such as IL-10 and IL-13, thereby suppressing intestinal inflammation and promoting mucosal healing (91). Similarly, activation of free fatty acid receptors FFAR1 and FFAR4 reduced lipid accumulation by enhancing fatty acid metabolism and induced M2 macrophage polarization, concomitantly increasing the expression of CD206, carnitine palmitoyltransferase-1α (CPT-1α), and anti-inflammatory cytokines (IL-4, IL-10, IL-13), ultimately ameliorating DSS-induced colitis (92). Concurrently, activation of the IL-4–STAT6 signaling pathway promoted M2 polarization and improved colonic mucosal injury (93), while this process can be suppressed by certain chemokines (94). M2 macrophages release anti-inflammatory mediators such as IL-10 and TGF-β, along with extracellular matrix (ECM) components, which collectively support epithelial repair and tissue remodeling (95). Additionally, they contribute to regenerative processes (96), mediated in part by hepatocyte growth factor (HGF) (97, 98), and initiate reparative mechanisms through pathways involving formyl peptide receptor (FPR)/annexin A1, NADPH oxidase 1 (NOX1), or IL-10/CREB/WISP-1 signaling (99). When exposed to microbial stimuli, M2 macrophages generate TNF-α, which triggers epithelial NF-κB activation, a critical regulator of mucosal homeostasis and inflammatory control (100). Although these cells predominantly display an M2-like phenotype, which appears essential for mucosal healing, their precise role in UC pathogenesis requires further investigation.

Under homeostatic conditions, tolerogenic macrophages are induced by dietary antigens or commensal microbiota, exhibiting a non-inflammatory profile characterized by diminished pro-inflammatory cytokine secretion and nitric oxide production, thereby preserving mucosal equilibrium (101). Following tissue damage, colonic macrophages engage in phagocytic clearance of pathogens and apoptotic cells, supporting microbial defense and epithelial repair. In UC, M2-polarized macrophages demonstrate dual functionality, combining antimicrobial activity and tissue remodeling with anti-inflammatory cytokine release, thereby alleviating intestinal injury (77). Emerging research has identified vessel-associated macrophages (VAMs) localized near colonic blood vessels. Single-cell transcriptomic analyses reveal elevated expression of genes associated with angiogenesis in these cells (102), with features aligning with M2 phenotype. VAMs contribute to a gut-vascular barrier, preventing microbial translocation to liver/systemic circulation (103, 104), effectively serving as vascular sentinels that safeguard microbial containment and vascular stability. Current therapeutic approaches targeting macrophage biology in UC focus predominantly on cytokine signaling modulation and polarization state manipulation (105).

## 5 The dynamic crosstalk between neutrophils and macrophages in UC

The pathogenesis of UC involves a complex interplay between neutrophils (NEUs) and macrophages, wherein neutrophil-derived mediators, including cytokines and neutrophil extracellular traps (NETs), modulate macrophage behavior (106, 107). IL-1β, TNF-α, and IL-6 released by activated NEUs promote macrophage polarization toward the pro-inflammatory M1 phenotype (108). Consequently, these polarized macrophages enhance the inflammatory response by producing additional cytokines and recruiting more immune cells to damaged tissues, exacerbating mucosal injury and perpetuating disease progression (12). M1 macrophages, in turn, secrete inflammatory cytokines such as IL-6 and IL-12, amplifying mucosal injury (109). NETs contribute to amplify the inflammatory cascade by reinforcing inflammatory signaling and providing a structural framework that facilitates macrophage infiltration (110, 111). However, emerging evidence suggests that NETs and neutrophil-derived signals may also play a role in resolving inflammation. In specific contexts, NETs promote the polarization of macrophages toward an M2 phenotype, characterized by the release of anti-inflammatory cytokines like IL-10 and TGF-β, which facilitate tissue repair (112). This dual functionality of NETs and NEUs underscores the intricate nature of their interactions with macrophages in UC. Given their opposing roles in inflammation and repair, targeting these cellular dynamics may present novel therapeutic opportunities for disease management.

## 6 Conclusion

The pathogenesis of UC is intricately linked to the dysregulated activities of NEUs and macrophages, which collectively drive inflammation, tissue injury, and impaired healing. NEUs amplify mucosal damage via ROS, NETs, and proteolytic enzymes. However, the protective subsets of NEUs, alongside their reparative cytokines, demonstrate their functional duality. Similarly, macrophages exhibit context-dependent roles: M1 polarization perpetuates inflammation through cytokine storms and barrier disruption, while M2 phenotypes promote microbial defense and epithelial repair.

To achieve histologic remission, which remains the gold standard for UC treatment, targeted therapies directed at NEUs and macrophages must be tailored to individual patient profiles. Specifically, patients with persistent subclinical inflammation despite endoscopic healing may benefit from therapies that more precisely modulate neutrophil activity, such as NET and MMP-9 inhibitors, or macrophage polarization strategies that encourage a shift toward the M2 phenotype. By focusing on these strategies, we may overcome challenges related to therapeutic resistance and the heterogeneity of UC, ultimately improving long-term patient outcomes. Further research into immune-stromal crosstalk and novel therapeutic agents is essential to refine treatment protocols for UC and move toward personalized, immune-centric approaches that can address the underlying mechanisms of persistent disease.
